# Relative power and sample size analysis on gene expression profiling data

**DOI:** 10.1186/1471-2164-10-439

**Published:** 2009-09-17

**Authors:** M van Iterson, PAC 't Hoen, P Pedotti, GJEJ Hooiveld, JT den Dunnen, GJB van Ommen, JM Boer, RX Menezes

**Affiliations:** 1 Center for Human and Clinical Genetics, Leiden University Medical Center, Leiden, the Netherlands; 2 Netherlands Bioinformatics Centre, Nijmegen, the Netherlands; 3 Nutrigenomics Consortium, TI Food and Nutrition, Wageningen, The Netherlands; 4 Nutrition, Metabolism and Genomics Group, Division of Human Nutrition, Wageningen University, Wageningen The Netherlands; 5 Leiden Genome Technology Center, Leiden University Medical Center, Leiden, the Netherlands; 6 Laboratory of Pediatrics Erasmus Medical Center, Sophia Children's Hospital, Rotterdam, The Netherlands; 7 Department Epidemiology and Biostatistics, VU Medical Center, Amsterdam, the Netherlands

## Abstract

**Background:**

With the increasing number of expression profiling technologies, researchers today are confronted with choosing the technology that has sufficient power with minimal sample size, in order to reduce cost and time. These depend on data variability, partly determined by sample type, preparation and processing. Objective measures that help experimental design, given own pilot data, are thus fundamental.

**Results:**

Relative power and sample size analysis were performed on two distinct data sets. The first set consisted of Affymetrix array data derived from a nutrigenomics experiment in which weak, intermediate and strong PPAR*α *agonists were administered to wild-type and PPAR*α*-null mice. Our analysis confirms the hierarchy of PPAR*α*-activating compounds previously reported and the general idea that larger effect sizes positively contribute to the average power of the experiment. A simulation experiment was performed that mimicked the effect sizes seen in the first data set. The relative power was predicted but the estimates were slightly conservative. The second, more challenging, data set describes a microarray platform comparison study using hippocampal *δ*C-doublecortin-like kinase transgenic mice that were compared to wild-type mice, which was combined with results from Solexa/Illumina deep sequencing runs. As expected, the choice of technology greatly influences the performance of the experiment. Solexa/Illumina deep sequencing has the highest overall power followed by the microarray platforms Agilent and Affymetrix. Interestingly, Solexa/Illumina deep sequencing displays comparable power across all intensity ranges, in contrast with microarray platforms that have decreased power in the low intensity range due to background noise. This means that deep sequencing technology is especially more powerful in detecting differences in the low intensity range, compared to microarray platforms.

**Conclusion:**

Power and sample size analysis based on pilot data give valuable information on the performance of the experiment and can thereby guide further decisions on experimental design. Solexa/Illumina deep sequencing is the technology of choice if interest lies in genes expressed in the low-intensity range. Researchers can get guidance on experimental design using our approach on their own pilot data implemented as a BioConductor package, SSPA .

## Background

Genome-wide technologies such as microarray and sequencing are intensively used to study differential expression in e.g. disease and/or treatment, often compared with controls. Power and sample size analysis give valuable information about the performance of the experiment: what is the optimal number of replicates? Is the power sufficient to detect a biological effect?

Absolute power and sample size estimation must be done using pilot experimental data in each problem separately, as they are influenced by variability that is both technical as well as biological [1]. For this a technology must be chosen. So it is important to understand beforehand how relative power and sample size behave depending on the technology used. In particular, different technologies may display different power depending on the gene expression range.

Here we focus on estimating relative change in power and sample size, given either different effect sizes or different expression profiling technologies. In each case results are derived from pilot experiments, so conclusions relate directly to practice. The different expression profiling technologies include commercial and home-spotted gene-expression microarray platforms, as well as a deep sequencing technology. For power and sample size calculations, we adapted the method proposed by Ferreira *et al*. [2].

## Methods

### Power and sample size estimation

Consider the case where samples are studied under two conditions, and interest lies in finding genes differentially expressed between these conditions. We use the power and minimal sample size calculation method of Ferreira *et al*. [2]. This method assumes that for a set of test statistics measuring the differential expression, their distributions are given as each having a normal distribution (*μ*, *σ*^2^). For each gene, under the null hypothesis *H*_0 _of non-differential expression we have the mean *μ *= 0, and under the alternative *H*_1 _of differential expression *μ *≠ 0. If we represent by *K, L *the cumulative distribution functions (CDF) of the test statistics under *H*_0 _and under *H*_1_, respectively, then the observed test statistics have mixture CDF *M *given by

(1)

where *λ *represents the density of effect sizes *θ *and *π*_0 _the proportion of non-differentially expressed genes.

Effect sizes can be seen as the difference between a gene's mean expression levels at two conditions, divided by its pooled standard deviation. Note that *M *is observed and *K *and *L *are given, so *π*_0 _and *λ *need to be estimated. After estimating *π*_0 _using the approach suggested by Langaas et al. [3], *λ *is estimated by a deconvolution estimator. The average power can be estimated by solving the following equation for *u*:

(2)

where Γ represents the power for a single gene as function of the p-value *u *and effect size *θ *and *δ *is the user-defined false discovery rate. In fact, Ferreira *et al*. [2] showed that the average power, given by equation 2, is controlled for multiple testing through the adaptive Benjamini-Hochberg method [4]. This is essentially the same as the originally proposed false discovery rate method of Benjamini and Hochberg [5], corrected by the proportion of differentially expressed genes to avoid over-estimation.

The effect size density is estimated unconstrained, so after it is obtained it must be constrained to being non-negative, whilst integrating to 1. To avoid discontinuities where the constraint is applied, we re-adjust the *π*_0 _estimate (see Additional file 1).

Note that to estimate the average power in this way involves also the power to detect effect sizes around zero, which are technically very difficult to measure accurately. A small region around zero can be defined that will be excluded from the density of effect sizes and thereby increases the estimated average power.

### Data Description

#### Simulation experiment

A simulation study was performed based on the simulation perviously described by Langaas *et al*. [3]. While keeping the proportion of non-differentially expressed genes fixed at 0.8, we varied the effect size distribution. Specifically, three different effect size distributions were constructed based on a symmetric bitriangular distribution [3]. This means that each differentially expressed gene is either over- or under-expressed with equal probability, and that the mean effect size per over-expressed gene is chosen at random from values in a window between *a *= log_2_(1.2) and *b *>*a*, with the mode at *m *= log_2_(2), and for under-expressed genes a window between -*a *and -*b *is used, with the mode at -*m*. We used three values for *b*, namely log_2_(2), log_2_(4), log_2_(5), generating situations with weak, intermediate and strong effect sizes respectively. This means for example that the weak-effect situation has genes with effect sizes between log_2_(1.2) and log_2_(2).

A total of *N *= 250 simulations were performed with 20000 independently generated normalized expression values and *J *= 5 samples in each of two groups. Test-statistics were calculated as described by Langaas *et al*. [3]. The observed power was calculated as the number of differentially expressed genes with Benjamini-Hochberg FDR ≤ 10% (true positives), divided by *m*_1 _= 4000. In addition, the estimated power was evaluated using the method proposed by Ferreira *et al*. [6].

#### Example 1 - different biological effect sizes

PPAR*α *is a transcription factor that is activated upon binding by a variety of agonists, both of synthetic and natural origin [7]. In this experiment the outcome of specific PPAR*α *activation on murine small intestinal gene expression was examined using Affymetrix GeneChip Mouse 430 2.0 arrays. PPAR*α *was activated by several PPAR*α*-agonists that differed in activating potency. In this paper the data of three agonists were used, namely Wy14,643, fenofibrate and trilinolenin (C18:3). The first two compounds belong to the fibrate class of drugs that are widely prescribed to treat dyslipidemia, whereas trilinolenin is an agonist frequently found in the human diet. For intestinal PPAR*α*, Wy14,643 is the most potent agonist followed by C18:3 and fenofibrate, as is detailed elsewhere (Hooiveld *et al*. manuscript in preparation). Since time of exposure also affects the effect size, intestines were collected 6 hrs (all three agonists) or 5 days (Wy14,643 and fenofibrate only) after exposure. Details of the rationale behind these designs have been previously published [8,9]. An overview of the dataset is given in the upper part of table 1. Probesets were redefined according to Dai *et al*. [10]. Expression estimates of probesets were obtained by GC-robust multi-array (GCRMA) analysis, employing the empirical Bayes approach for background adjustment, followed by quantile normalization and summarization [11]. For each compound and exposure time, lists of moderated t-test statistics were computed, using the empirical Bayes linear regression model as implemented in limma[12], for each contrast representing the effect of compound in PPAR*α*-null mice compared to wild-type mice. Based upon these lists, which include the full range of effect sizes, the power and sample size analysis was performed.

**Table 1:** Overview of data sets in Example 1 and 2

**Experiment**	**Genes**	**group A**^**1**^	**group B**
strong, 6 hours	16539	4 (wild-type)	4 (null)
intermediate, 6 hours	16539	4 (wild-type)	5 (null)
weak, 6 hours	16539	5 (wild-type)	5 (null)
strong, 5 days	16539	4 (wild-type)	4 (null)
weak, 5 days	16539	4 (wild-type)	4 (null)

Affymetrix	45101	5 (wild-type)	5 (transgenic)
Agilent	41232	5 (wild-type)	5 (transgenic)
Illumina	46120	5 (wild-type)	5 (transgenic)
home-spotted	21771	5 (wild-type)	5 (transgenic)
Solexa/Illumina	34477	4 (wild-type)	4 (transgenic)

^1 ^The sample size given refer to the number of biologically independent samples.

#### Example 2 - different expression profiling platforms

Gene expression profiles in the hippocampi of transgenic *δ*C-doublecortin-like kinase mice were compared to wild-type. The sample size and power analysis was applied to expression data relating to the same samples, measured either by one of several microarray platforms, or by Solexa/Illumina deep sequencing [13].

In this analysis we included expression data for the same samples obtained with four microarray platforms, previously analyzed in the platform comparison study of Pedotti *et al*. [14], namely Affymetrix, Agilent, Illumina and home-spotted oligonucleotide arrays, the last containing the 22K Sigma-compugen collection. Ten microarrays were used for each platform. For the one-color platforms (Affymetrix and Illumina), each individual RNA was hybridized to one microarray, yielding five hybridizations in each of the groups wild-type and transgenic mice. A direct design was used for hybridization of the two-color arrays (Agilent and home-spotted), *i.e*. each microarray was hybridized with two RNA samples from different groups. Dye-swapped hybridizations were done with non-identical pairs. This design yielded ten hybridizations in each of the groups, five of which being biologically independent. The data has been pre-processed as described by Pedotti *et al*. [14]. mRNA expression levels from four samples of each group were also measured using the Solexa/Illumina deep sequencing; for details about sample preparation and pre-processing, see [13]. Moderated t-test statistics were calculated, for each technology, using the empirical Bayes linear regression model as implemented in limma[12].

To enable comparison between technologies, for each technology subsets were generated containing only genes mapped to the same Ensembl tags, leaving 9504 genes per technology [14]. The datasets were stratified into three subsets of genes of approximately equal size (3168) as follows. First the median-expression values across wild-type samples for the Solexa/Illumina data were calculated, per Ensembl tag. Then the 33rd and 67th percentiles of these values were obtained. These two values were used to determine three strata: tags with Solexa/Illumina median -expression below 33rd percentile were put into the low-intensity range, followed by tags with median-expression between 33th and 67th percentiles being put into the intermediate-intensity range, and finally remaining tags were put into the high-intensity range. In the power and sample size analysis a region near zero ([-0.5, 0.5]) was excluded from the density of effect sizes.

### Software

We used the R [15] environment for statistical computing for all calculations, the BioConductor [16] packages gcrma[11], limma[12] and multtest[17], qvalue[18] and for graphs we used the lattice[19] package. We adapted the original S-Plus scripts kindly made available to us by Ferreira [6] and developed an R package called SSPA, available from BioConductor [16].

## Results

### Simulation experiment

**Figure 1:**
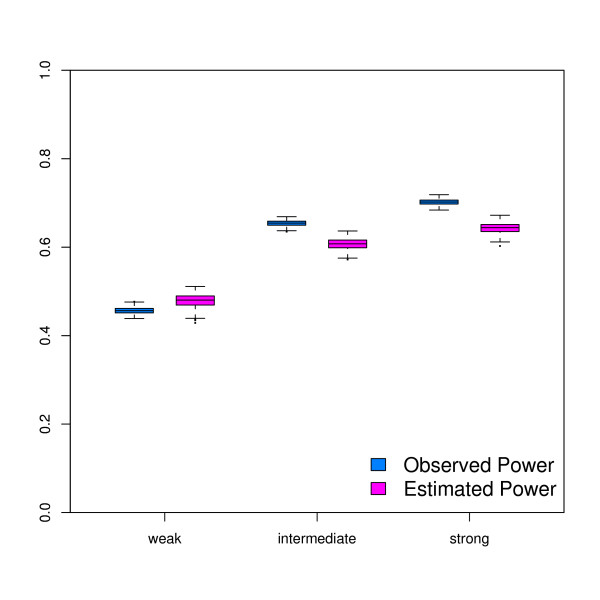
**Simulation Experiment: Compares estimated powerwith the true positive rate**. Box-and-whisker plot of the estimated average power and true positive rate for all the simulations, for the three different effect size distributions. The Power is displayed on the y-axis and the different simulation scenario's are displayed on the x-axis fromleft to right: weak-, intermediate- and strong-effect sizes respectively.

The main purpose of this small simulation experiment was to show that the proposed method of Ferreira *et al*. [2] is able to perform relative average power estimations in a scenario of varying effect size distributions. Although the estimated average power is slightly conservative, the relative power is in agreement with the true positive rate, the fraction of truly differentially expressed genes amongst the significant genes (figure 1).

#### Example 1 - different biological effect sizes

**Figure 2:**
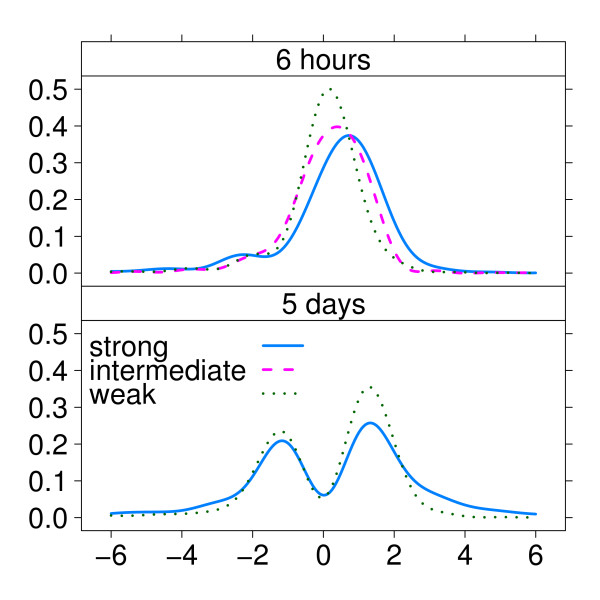
**Example 1: Estimated densities of effect sizes for combinations of compounds and exposure times**. The upper panel shows densities for 6 hours exposure time, the lower panel for 5 days exposure time, with on the x-axis the standardized effect size and on the y-axis the estimated densities. The different compounds Wy14,643 (strong-), C18:3 (intermediate-) and fenofibrate (weak-activating potency) correspond respectively to the solid(blue)-, dashed(pink)- and dotted(green)-lines.

In this first real-life example we provide a proof-of-principle that our method is able to correctly estimate relative power in a setting where different effect sizes are induced as a consequence of the treatment with compounds with different potency's and exposure times. This experiment was originally setup to examine the effects of PPAR*α*-activation by synthetic as well as nutritional agonists on gene expression in various tissues in mice, including intestine. Comparative analysis in PPAR*α*-null mice enabled the specific study of the regulatory role of this transcription factor. Previous, biology-driven analysis of the array data sets identified a hierarchy in PPAR*α*-activating potency [8] (Hooiveld *et al*., manuscript in preparation). Test statistics were obtained by measuring the differential response on gene expression between wild-type and PPAR*α*-null mice. We estimated power and minimal sample size for each combination of compound and exposure time separately, fixing the false discovery rate at 10%. Figure 2 shows the estimated densities of effect sizes, per compound and exposure time. These densities describe the standardized effect sizes among the differentially expressed genes. For each exposure time, the densities show increasingly heavier tails with the expected PPAR*α*-activating potency of the compounds, from the weakest (fenofibrate) to the strongest (Wy14,643). Moreover, for all compounds there is relatively more up-regulation than down-regulation. When comparing exposure times (upper and lower panels), it is obvious that longer exposure yields more pronounced effects, represented by the bimodal densities, compared with shorter exposure, where densities representing the mixture of up- and down-regulation on genes display a single overlapping mode, with a subtle separate effect seen on the lower tail for the stronger compounds.

From the left-panel of table 2 we can see that, by increasing exposure time from six hours to five days, the estimated power was doubled for the more potent compound Wy14,643, and increased almost by a factor of four for the weaker compound fenofibrate. Indeed, the Wy14,643 reached around 60% power, where the fenofibrate had just 40% power. In addition, as the compound got less potent, the proportion of differentially expressed genes decreased, as expected. So it is clear that less potent compounds, or the ones exposed for a shorter time, yield little power to detect differences.

**Table 2:** Estimated power and adjusted proportions of non-differentially expressed genes for the pilot studies.

**Example 1**			**Example 2**		
**Experiment**	**Power**	***π***_**0**_	**Technology**	**Power**	***π***_**0**_

strong, 6 hours	0.27	0.68	Affymetrix	0.32	0.81
intermediate, 6 hours	0.10	0.81	Agilent	0.35	0.65
weak, 6 hours	0.10	0.76	Illumina	0.20	0.84
strong, 5 days	0.60	0.51	Home-Spotted	0.18	0.68
weak, 5 days	0.38	0.73	Solexa/Illumina	0.51	0.52

The power is estimated with a 10% FDR.

**Figure 3:**
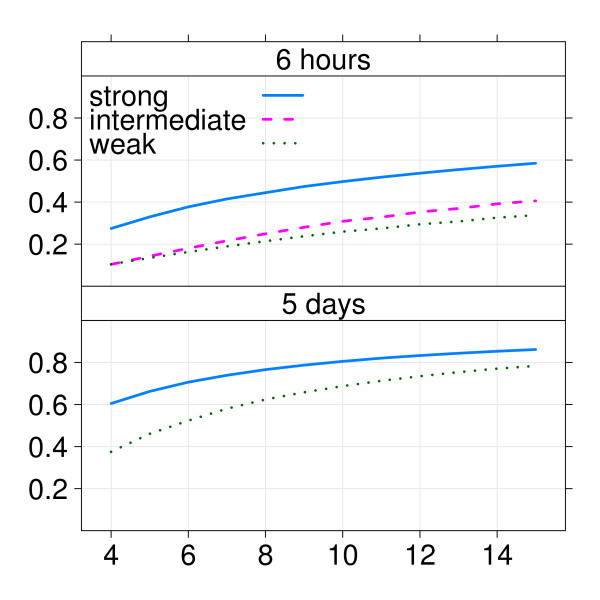
**Example 1: Power curves for the different combinations of compounds and effect sizes**. The upper panel shows the power for 6 hours exposure time, the lower panel the 5 days exposure time, with on the x-axis the sample size and on the y-axis the estimated power using a 10% FDR. The different compounds Wy14,643 (strong-), C18:3 (intermediate-) and fenofibrate (weak-activating potency) correspond respectively to the solid(blue)-, dashed(pink)- and dotted(green)-lines.

A more complete picture of the variation in power as the sample size increases is given in figure 3, for each combination of compound and exposure time. As expected, the average power increased with PPAR*α*-activating potency and with exposure time. For example, if a power of 50% was desired to study the strong Wy14,643 compound, 10 arrays per group would be necessary considering a six hours exposure, whilst the same power would be already achieved using a five days exposure with four arrays per group. The other two compounds needed more than 15 samples per group for a six hours exposure to yield the same power, which after a five days exposure would be achieved with the fenofibrate compound with six samples per group.

#### Example 2 - different expression profiling platforms

This study was designed to explore the capabilities of expression profiling technology to identify subtle differences in gene expression. To estimate power, it is essential to estimate the proportion of non-differentially expressed genes (*π*_0_). In table 2 (right-panel) we report *π*_0 _for the different expression profiling platforms, as estimated by the method of Langaas *et al*. [3]. We used alternative methods for estimation of *π*_0 _with essentially similar results (see Additional file 2). The estimated proportion of non-differentially expressed genes differ significantly between the different technologies, with Illumina displaying the highest proportion (right-panel of table 2). However, perhaps the most marked point is that the power is generally low for all technologies used. This is a reflection of the fact that the effects on expression are mostly subtle.

**Figure 4:**
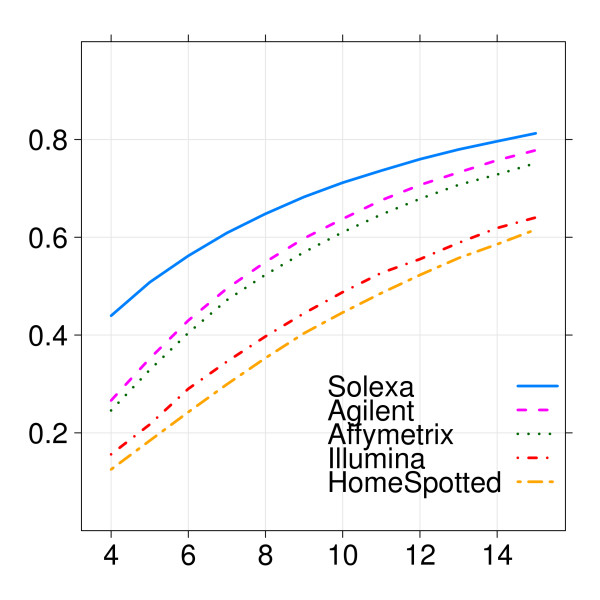
**Example 2: Power curves for the different expression profiling platforms**. The five curves solid(blue), short-dashed(pink), dotted(green), dot-dashed(red) and two-dashed(orange) correspond to Solexa/Illumina, Agilent, Affymetrix, Illumina, and home-spotted oligonucleotide arrays respectively. On the x-axis the sample size is displayed and on the y-axis is the estimated power using a 10% FDR.

As before, a better overview is given by the estimated power curves in figure 4. The power continued to be generally low for the sample sizes considered, but increased with sample size. Here Solexa/Illumina, Agilent and Affymetrix are shown to be the best performing technologies for all sample sizes. The higher performance of Agilent compared to Affymetrix may be a consequence of the pilot study size, upon which the estimation of the power curve depends. As the number of samples differs per technology (one-color: 5 biological replicates per group; two-color: 5 biological replicates, each with 2 technical replicates; and Solexa/Illumina deep sequencing: 4 biological replicates), there is considerably more certainty about estimates for Agilent and home-spotted, since their technical variability is better estimated, than for the other cases, especially so for Solexa/Illumina with the smallest sample size. Yet, for all sample sizes Solexa/Illumina was estimated as having the highest power, which is an indication of how precise and powerful this technology is.

Considering the two one-color platforms, Illumina yields less power than Affymetrix in this experiment (see right-panel of table 2). This is also reflected by the estimated densities of effect sizes (not shown).

**Figure 5:**
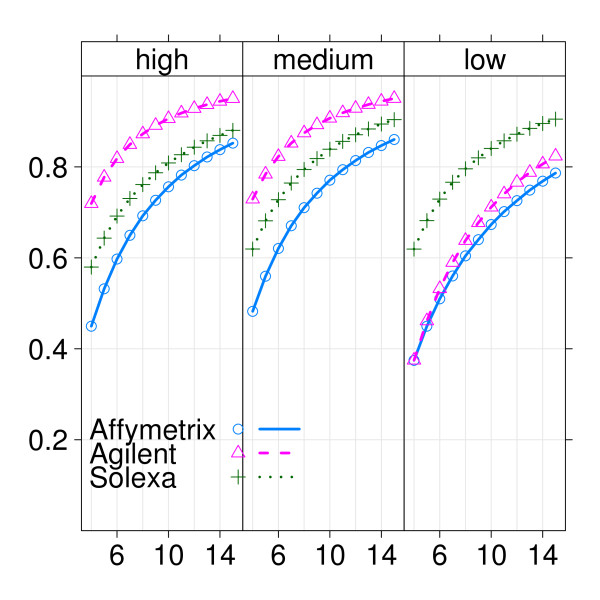
**Example 2: Power curves for three technologies and three intensity ranges (ordered by intensity range)**. The three panels show power curves for Affymetrix, Agilent and Solexa/Illumina, with on the x-axis the sample size and on the y-axis the estimated power using a 10% FDR.

**Figure 6:**
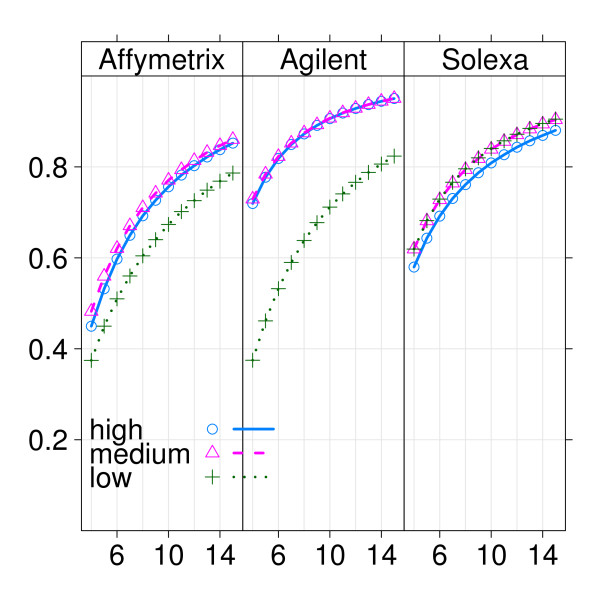
**Example 2: Power curves for three technologies and three intensity ranges (ordered by platform)**. The three panels show power curves for Affymetrix, Agilent and Solexa/Illumina, with on the x-axis the sample size and on the y-axis the estimated power using a 10% FDR.

The largest effects in this experiment were observed for genes expressed in the low-intensity range, so technologies that are more sensitive to detect differences in this range are expected to perform better. In order to gain more insight into the sensitivity of the various technologies, we split genes into subsets according to them being expressed in the low-, medium- and high-intensity ranges, as given by wild-type median- expression ranking of Solexa/Illumina. For each technology and subset the power was estimated again for a range of sample sizes. Results are displayed in figures 5 and 6 for Affymetrix, Agilent and Solexa/Illumina, where figure 5 is ordered by intensity range and figure 6 is ordered by platform. In the low-intensity range, Solexa/Illumina displays considerably more power than the two microarray platforms, whilst in medium- and high-intensity ranges Affymetrix and Solexa/Illumina were comparable, with Agilent having a higher power (figure 5). More interestingly perhaps, Solexa/Illumina displays comparable power across the three intensity ranges, whilst each one of the microarray platforms displays markedly less power in the low-intensity range compared to the intermediate- and high-intensity ranges (figure 6). This is due to the presence of background, which is absent in Solexa/Illumina, but affects measurements in the low-intensity range of microarray platforms. So the largest effects found in the low-intensity range in this study are duly detected by Solexa/Illumina, but missed by the microarray platforms. This is further exemplified in Additional file 3, where the same comparison is made without filtering for small effect sizes.

## Discussion

We aim at helping researchers to understand: a) how much change in sample size different expression profiling technologies require to yield the same power; and b) by how much the sample size required for a fixed power would change, if smaller effects were to be detected. In order to do so, we use experiments carefully designed to answer these questions. Our experiments use tissue samples from animal models, commonly used in practice. Not only our conclusions are easily applicable to the design of microarray experiments, but also our approach is available for researchers to use on their own data via our BioConductor package SSPA.

For our study, we use the method proposed by Ferreira *et al*. [2]. It makes use of a pilot study to estimate distribution of effect sizes and proportion of non-differentially expressed genes, based upon which power is estimated.

Various other methods to estimate sample size and power in microarray studies have been proposed. The first few assumed that multiple testing correction is done via control of the familywise error rate [20,21], which is unlikely to be the case in current practice. More recently, methods were proposed to handle the more commonly used control of false discovery rate [22-25,2]. The methods differ in how they treat the distribution of effect sizes; simpler methods assumed a fixed value for all differentially expressed genes, or took a subset of the largest effect sizes [22,25]. These are unlikely to correctly describe effects from experimental studies. Recently proposed methods [2,24,26] estimated the distribution of effect sizes from a pilot data set. Ferreira [2] assumes that the test statistics follow a normal distribution, which is unlikely to be the case. However, the extension of Ferreira's method to consider statistics with a more suitable Student-t distribution is not trivial. Indeed, to solve equation 2 is a much harder analytical problem under the Student-t distribution. Jørstad *et al*. [24] proposed to solve this problem by discretizing the effect sizes and then estimating the components of the resulting mixture. Ruppert *et al*. [26] proposed to estimate the density of effect size by a linear combination of splines optimized via penalized least squares. The number of parameters that needs to be estimated by both methods is considerably larger than by the method of Ferreira *et al*. [2], making them computationally much more intensive. For this reason, we chose to use Ferreira's method.

The simulation experiment shows that the estimated power is in agreement with the observed power. Important for the power estimation is the estimation of the proportion of non-differentially expressed genes. The *π*_0 _estimates of the simulation experiment were all conservative and a little less than *π*_0 _= 0.8, which may have led to overestimation of the power. However, the opposite was observed: the estimated power was found to be conservative. Generally speaking it is good to be on the conservative side in power calculations. However, this may not be the case in other applications. Indeed, we have observed that the relationship between observed and estimated power depends on both *π*_0 _and the effect size distribution (data not shown). This issue deserves further investigation but is beyond the scope of this paper.

Both our simulation study as well as example 1 demonstrate that our method correctly ranks the power of different experimental scenarios and is thus suited to evaluate the relative capacity to identify differentially expressed genes. Some of the results obtained with this method are as expected: in example 1, potent compounds yield higher power than weaker compounds, as does longer exposure time compared to a shorter one (example 1). The hierarchy of PPAR*α*-activating compounds found with this method confirms previously biologically-driven analysis [8] (Hooiveld *et al*. manuscript in preparation). However, some results are unexpected, such as that the power seems to increase little after a certain sample size (12, for 5-days exposure). Since the power for 12 samples per group is already acceptably high (80% for the stronger compound, over 70% for the weaker one), this result suggests that it is useless to analyse more than 12 samples to find differentially expressed genes.

The higher power for the longer-exposed compounds is associated with a markedly bimodal density of effect sizes (figure 2). This density represents additional and important information for the researcher. In this case, for example, it can be seen that there are more genes up- regulated than down-regulated after short-term exposure, and that after long-term exposure this is more balanced, suggesting that up-regulation is kicked off earlier on. So, we have shown how the density of effect sizes reflects varying distributions of differential expression. This is intuitive and seems trivial, but no other method has produced this result before [6].

Example 2 shows great difference in performance for different expression profiling technologies to detect a subtle biological effect. Commercial microarray platforms perform better than the home-spotted mainly due to their higher reproducibility. The poor performance of Illumina may be attributed to the fact that less probes are used than Affymetrix. By estimating power separately for genes expressed in intensity ranges varying from low to high, we can clearly see the added value of the Solexa/Illumina deep sequencing technology compared with microarrays. Due to background, microarray platforms often cannot reliably measure expression in the low-intensity range. Indeed, Solexa/Illumina displays with 4 replicates per group the same power as Agilent and Affymetrix with 7 replicates per group (figure 4). The reduced power of microarray technologies is shown to be mainly due to lack of power to detect differential expression for lowly expressed genes, possibly due to presence of background intensities, a problem that does not affect Solexa/Illumina.

The estimated proportion of non-differentially expressed genes *p*_0 _shows great difference between the different expression profiling technologies. This is likely due to differences in genome mapping [14] and hybridization efficiency. Since it is known that the same samples were hybridized to the different platforms, one might wonder if by using a common, fixed *π*_0_, value for all platforms more consistent results would have followed. That is not the case: power estimates are robust to variations of roughly 10% around the estimated *π*_0 _value (data not shown). Moreover, we believe this should not be done, as effects of technical differences between the platforms would then have been ignored, which is undesirable. So, we believe that for each experiment *π*_0 _should be estimated from the data, and that a sensitivity analysis may help reassure the researcher that power estimates are reliable.

Our example 2 is not trivial and no previous article, to our knowledge, has produced power and sample size calculations in such datasets. Indeed, the MAQC study [27,28] only involves technical replicates. So we do believe our work, by involving not only 4 different microarray platforms, but more importantly also a deep sequencing technology, does yield new knowledge. In particular, no previous work has shown that deep sequencing technology displays more power than microarrays in the presence of biological, in addition to technical, variability.

The objective of the study plays an important role in choosing the FDR-control level. Indeed, if results are meant to be further explored via high-dimensional tools such as pathway analysis, then there is interest in having a longer list of possibly interesting features, albeit with a larger FDR. This effectively expands the space on which the subsequent analysis tool will look for associations, improving the chance of finding more subtle, and for that less obvious, ones. If, on the other hand, the list of selected features must be validated by time-consuming and labour intensive techniques, then a shorter list obtained with as low an FDR as possible is the best. For our objectives, we needed only to have at least a few features selected at each instance in order to be able to draw comparisons, but preferably not too many, and we find this is achieved by controlling the FDR at 10%. We made a fast and easy-to-use implementation of a power and minimal sample size calculation method adapted from Ferreira *et al*. [2]. The only input needed is a set of test statistics obtained from the pilot data and the number of samples of the two groups. More details about this R package will appear elsewhere.

## Conclusion

In conclusion relative power and sample size analysis can help researchers make important decisions about technology used for gene expression profiling. We showed that if interest lies in genes expressed in the low-intensity range Solexa/Illumina deep sequencing is the superieur technology compared to microarray technology. Furthermore, we have implemented our method via a BioConductor package so that other researchers can use it on their own pilot data.

## Authors' contributions

MI performed the data analysis. PACH provided the platform comparison data and interpretations of the results, and contributed to the manuscript. PP performed the differential gene expression analysis of the platform comparison. GJEJH provide the nutrigenomics data and contributed to the manuscript. JTD was involved in many useful discussions. GJBO actively supported the deep sequencing experiments. JMB was involved in study design and interpretation of results. MI and RXM wrote the manuscript. All authors read and approved of the final manuscript.

## Supplementary Material

Additional file 1 **Adjusting the proportion of non-differentially expressed genes**. A description of how the method for adjusting the the proportion of non-differentially expressed genes works. Click here for file

Additional file 2 **Estimates of proportion of non-differentially expressed genes (*π*_0_) using three different methods (Langaas, Storey and Ferreira), as well as estimates of the power**. This table enables comparison between the methods used to compute *π*_0_, and their effect on power. Click here for file

Additional file 3 **Example 2: Power curves for three technologies and three intensity ranges**. The figure has three panels which show power curves for high-, medium-, low-intensity range, with on the x-axis the sample size and on the y-axis the estimated power using a 10% FDR. Each panel shows the power as function of the sample size for three technologies Affymetrix (solid, blue), Agilent (short-dashed, pink) and Solexa/Illumina dotted green). These plots were obtained without the exclusion of a small region around zero. Click here for file
