# Supplementary material for: Relative power and sample size analysis on gene expression profiling data
Source: BMC Genomics. 2009 Sep 17;10:439. doi: 10.1186/1471-2164-10-439 (PMC2759969; doi:10.1186/1471-2164-10-439)
Supplement: Additional file 1 — Adjusting the proportion of non-differentially expressed genes. A description of how the method for adjusting the the proportion of non-differentially expressed genes works. [file 1471-2164-10-439-S1.PDF]

Adjusting the proportion of non-differentially expressed genes

In order to make realistic power and sample size calculations the distribution of effect sizes needs to be known. A few authors [1–3] have proposed methods to estimate the distribution of effect size from pilot data, respectively using a deconvolution estimator, expectation-maximization algorithm or a spline-model. We use the deconvolution estimator [1]. The proportion of non-differentially expressed genes, π_0, is the first quantity to be estimated. The empirical density of the test statistics, m, is estimated from the set of test statistics from the pilot data.

The deconvolution involves solving the following equation for λ

$$\frac{m(t) - \pi_0 \phi(t)}{1 - \pi_0} = \int_{-\infty}^{+\infty} \phi\left(t - \theta\sqrt{N}\right) \lambda(\theta)\, d\theta, \quad (1)$$

where λ is the density of effect sizes, ϕ represents the density of the test statistics under H_0 and θ represents the effect size. The left-hand side of Equation 1 expresses the difference between the observed density of test statistics m and that of the assumed density of test statistics under H_0 (standard Normal, ϕ), weighted by the proportion of non-differentially expressed genes. This linear combination of densities puts certain constraints on the value of π_0. The constrain can be formulated as:

$$\min_i \left(m(t_i) - \pi_0 \phi(t_i)\right) \geq 0 \Rightarrow \pi_0 \leq \min_i \left(\frac{m(t_i)}{\phi(t_i)}\right). \quad (2)$$

This constraint is used to adjust the value of π_0 in order to guarantee that the estimated density of effect size is continuous and non-negative.

References

1. Ferreira J, Zwinderman A: **Approximate Power and Sample Size Calculations with the Benjamini-Hochberg Method**. *International Journal of Biostatistics* 2006, **2**.
2. Jørstad T, Midelfart H, Bones A: **A mixture model approach to sample size estimation in two-sample comparative microarray experiments**. *BMC Bioinformatics* 2008, **9**(117).
3. Ruppert D, Nettleton D, Hwang J: **Exploring the information in p-values for the analysis and planning of multiple-test experiments.** *Biometrics* 2007, **63**(2):483–95.

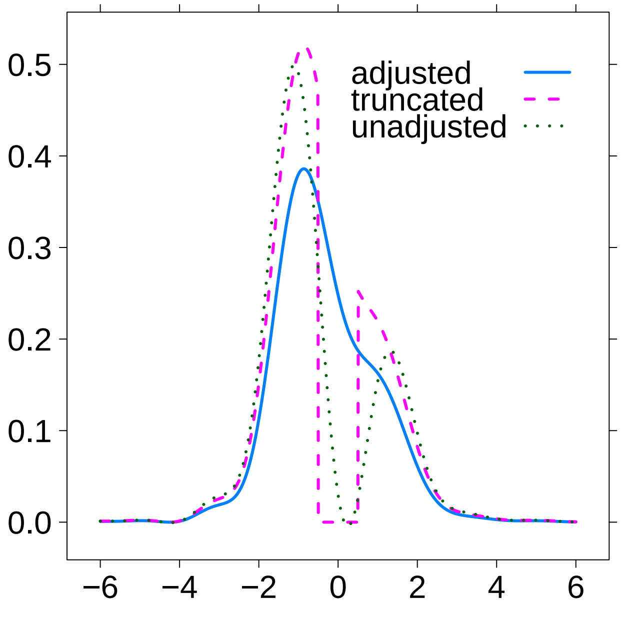

Adjustments of density of effect sizes: The three curves solid(blue)-, short-dashed(pink)-, dotted(green)-lines represent respectively the adjusted density, a density truncated near zero and the unadjust non-valid density. On the x-axis is the standardized effect size, and on the y-axis is the estimated density.
