# Supplementary material for: Relative power and sample size analysis on gene expression profiling data
Source: BMC Genomics. 2009 Sep 17;10:439. doi: 10.1186/1471-2164-10-439 (PMC2759969; doi:10.1186/1471-2164-10-439)
Supplement: Additional file 2 — Estimates of proportion of non-differentially expressed genes (π0) using three different methods (Langaas, Storey and Ferreira), as well as estimates of the power. This table enables comparison between the methods used to compute π0, and their effect on power. [file 1471-2164-10-439-S2.PDF]

Estimates of proportion of non-differentially expressed genes (π_0) using three different methods (Langaas, Storey and Ferreira), as well as estimates of the power computed with the adjusted π_0 that yield continuous and non-negative densities (see Additional file 1).

	power			π_0					
	Lang.	Stor.	Ferr.	Lang.		Stor.		Ferr.	
Affymetrix	0.31	0.31	0.30	0.76	0.61[1]	0.75	0.61[1]	0.59	0.56[1]
Illumina	0.19	0.19	0.19	0.83	0.58	0.83	0.58	0.67	0.58
Agilent	0.44	0.44	0.54	0.48	0.37	0.46	0.37	0.22	0.22
HomeSpotted	0.19	0.19	0.20	0.64	0.40	0.62	0.40	0.41	0.37
Solexa	0.45	0.45	0.45	0.74	0.46	0.73	0.46	0.53	0.46

[1]the second column of each method represents the adjusted π_0.
