# Supplementary material for: Relative power and sample size analysis on gene expression profiling data
Source: BMC Genomics. 2009 Sep 17;10:439. doi: 10.1186/1471-2164-10-439 (PMC2759969; doi:10.1186/1471-2164-10-439)
Supplement: Additional file 3 — Example 2: Power curves for three technologies and three intensity ranges. The figure has three panels which show power curves for high-, medium-, low-intensity range, with on the x-axis the sample size and on the y-axis the estimated power using a 10% FDR. Each panel shows the power as function of the sample size for three technologies Affymetrix (solid, blue), Agilent (short-dashed, pink) and Solexa/Illumina dotted green). These plots were obtained without the exclusion of a small region around zero. [file 1471-2164-10-439-S3.PDF]

Example 2: Power curves for three technologies and three intensity ranges.

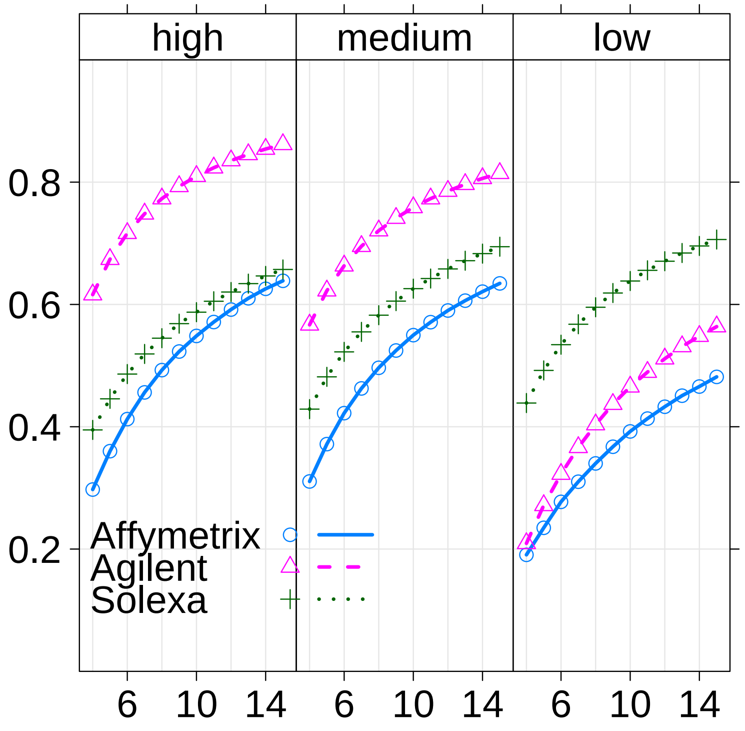

The three panels show power curves for high-, medium-, low-intensity range, with on the x-axis the sample size and on the y-axis the estimated power using a 10% FDR. Each panel shows the power curves as function of the sample size for three technologies Affymetrix (solid, blue), Agilent (short-dashed, pink) and Solexa/Illumina dotted green). These plots were obtained without the exclusion of a small region around zero, and show a slightly smaller power for Solexa/Illumina compared to when this small region is not excluded (figures 5 and 6). However, Solexa/Illumina still has superieur power to find differential expression in the low-intensity range.
